# Dynamic Range of Vertebrate Retina Ganglion Cells: Importance of Active Dendrites and Coupling by Electrical Synapses

**DOI:** 10.1371/journal.pone.0048517

**Published:** 2012-10-29

**Authors:** Rodrigo Publio, Cesar Celis Ceballos, Antonio C. Roque

**Affiliations:** Department of Physics, FFCLRP, University of Sao Paulo, Ribeirao Preto, Sao Paulo, Brazil; The Chinese University of Hong Kong, Hong Kong

## Abstract

The vertebrate retina has a very high dynamic range. This is due to the concerted action of its diverse cell types. Ganglion cells, which are the output cells of the retina, have to preserve this high dynamic range to convey it to higher brain areas. Experimental evidence shows that the firing response of ganglion cells is strongly correlated with their total dendritic area and only weakly correlated with their dendritic branching complexity. On the other hand, theoretical studies with simple neuron models claim that active and large dendritic trees enhance the dynamic range of single neurons. Theoretical models also claim that electrical coupling between ganglion cells via gap junctions enhances their collective dynamic range. In this work we use morphologically reconstructed multi-compartmental ganglion cell models to perform two studies. In the first study we investigate the relationship between single ganglion cell dynamic range and number of dendritic branches/total dendritic area for both active and passive dendrites. Our results support the claim that large and active dendrites enhance the dynamic range of a single ganglion cell and show that total dendritic area has stronger correlation with dynamic range than with number of dendritic branches. In the second study we investigate the dynamic range of a square array of ganglion cells with passive or active dendritic trees coupled with each other via dendrodendritic gap junctions. Our results suggest that electrical coupling between active dendritic trees enhances the dynamic range of the ganglion cell array in comparison with both the uncoupled case and the coupled case with cells with passive dendrites. The results from our detailed computational modeling studies suggest that the key properties of the ganglion cells that endow them with a large dynamic range are large and active dendritic trees and electrical coupling via gap junctions.

## Introduction

One of the many important features of the vertebrate retina is the capacity to respond to signals over a wide range of intensities with a dynamic range of several orders of magnitude [1], [2]. Individual neurons have limited dynamic ranges, so the large dynamic range of the retina must result from some interplay between single neuron characteristics and network structure and synaptic properties. This may be one the explanations for the diversity of cell types found in the retina and the complexity of its circuitry [3]–[6]. The ganglion cells in particular, which are the output cells of the retina and transmit information to higher brain regions in the form of action potentials that propagate along their axons, must have dynamic range enhancement mechanisms that prevent their firing rates from early saturation to preserve the dynamic range achieved at earlier stages in the retina [7]–[9].

A single neuron characteristic, which is claimed to be fundamental for enhancing the neuronal dynamic range in general is the size and complexity of the neuronal dendritic tree with active conductances [10], [11]. The idea behind this claim is that dendritic trees with many bifurcations and active ionic conductances act as *spatially extended excitable systems* whose nonlinear input-output transfer function endows the neuron with a large dynamic range. If this hypothesis is valid, most cells of the vertebrate retina do not benefit from this property because they have simple dendritic structures. The possible exceptions are the ganglion and amacrine cells, which are the most complex cells of the retina and have relatively intricate dendritic arbors [12], [13].

There is evidence in favor of a relationship between properties of ganglion cell dendritic trees and their firing behavior, however not exactly as predicted by the above theory. Computational studies with morphologically reconstructed models of ganglion cells of the salamander retina [12], [14] indicate that the branching complexity of the dendritic tree correlates only weekly with the electrophysiological response pattern and the input-output transfer function, measured by the firing rate *versus* stimulus current (F–I) curve. These studies found strong correlations between the total somatic plus dendritic surface area of the cell and its electrophysiological class and F-I curve.

From the point of view of network properties, a mechanism that may contribute to enhance the collective dynamic range of the network is electrical coupling between cells via gap junctions [15]–[17]. The coupling would increase sensitivity to weak stimuli while avoiding early saturation by nonlinear self-limiting mechanisms. This mechanism may be important for the vertebrate retina, since there is extensive evidence for electrical coupling between different cell types in the retina via connexin36 gap junctions [5], [6], [9], [18], [19]. Indeed, a biophysically detailed model of the rod pathways in the vertebrate retina has shown that electrical synapses between rods and AII amacrine cells may increase the dynamic range of the system [20].

Ganglion cells of the vertebrate retina are coupled by electrical synapses via dendrodendritic gap junctions [21]–[23]. In the spirit of the theory mentioned above [15]–[17], this may be interpreted as a means to enhance the collective dynamic range of the ganglion cells. However, to our best knowledge, no one has yet investigated the role of dendrodendritic gap junctions between ganglion cells on the dynamic range of the network comprised of them, especially with cell models that take into account dendritic morphology.

In this work we use morphologically reconstructed, multicompartmental models of ganglion cells of the vertebrate retina with realistic distributions of ion channels to perform two computational studies on the dynamic range of ganglion cells. The first is concerned with the dynamic range of isolated ganglion cells and its objective is to compare the effects of active and passive dendrites on the cells’ dynamic range and to assess what measure of size of dendritic arbor correlates better with the ganglion cell dynamic range: total dendritic surface area or number of dendritic branches. We measure the correlation between dynamic range and these two measures of dendritic tree size for a population of cell models with either passive or active dendrites. Our results show that active dendrites enhance the dynamic range in comparison with passive dendrites and when dendrites are active the dynamic range of isolated ganglion cells is positively correlated with either measure of dendritic tree size, though more strongly with total dendritic surface area than with number of dendritic branches.

The second study is aimed at assessing the role of ganglion cell coupling by gap junctions on the dynamic range of the ganglion cell population. We construct a network of ganglion cells by coupling them via dendrodendritic gap junctions with realistic conductance values. The network simulates a small area of the ganglion cell layer. We consider different configurations of the network, with passive/active dendrites and different values of the electrical synapse conductance. The dynamic range of the network is measured either directly, by the average firing rate of all neurons in the network, or indirectly, by the firing rate of a lateral geniculate nucleus pyramidal neuron model coupled by chemical synapses to all ganglion cells in the network. As far as we know, this is the first computational investigation of the dynamic range of a neural cell layer using reconstructed neurons with full morphologies and realistic ion channel distributions. Our results show unequivocally that electrical coupling, especially when dendrites are active, increases the dynamic range in comparison with the uncoupled case.

Our two results put together imply that to maximize the dynamic range of a population of vertebrate ganglion cells the best configuration would be cells with large and active dendritic trees coupled by gap junctions.

## Results

### Dynamic range of isolated ganglion cells

We worked with a sample of 20 morphologically and biophysically detailed models of ganglion cells from the tiger salamander (for details see Methods). Cells belonged to four different morphological groups (5 per group), based on the size and complexity of their dendritic trees [24]: medium-complex (MC), medium-simple (MS), small-complex (SC), and small-simple (SS).

**Figure 1:**
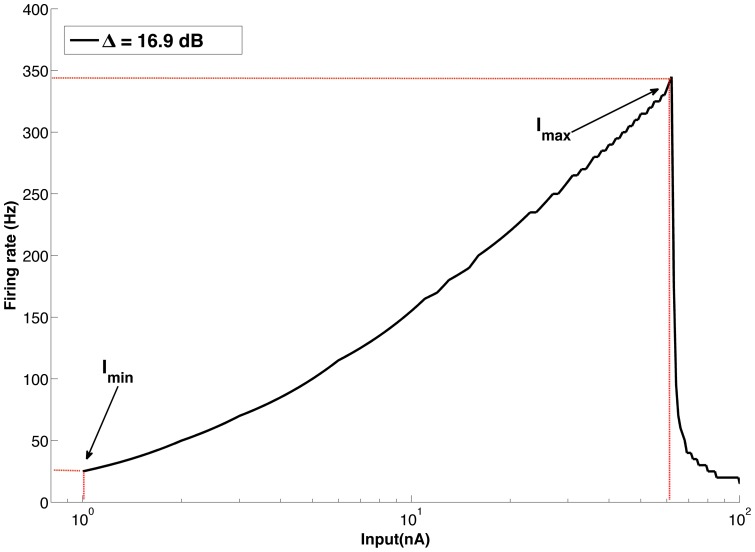
Response functions. Current clamp response for a sample cell from the SS group. The sampled cell has 47 branches and total dendritic area of 1284.67 μm2. (A) Voltage response to a current clamp of 10 pA amplitude. (B) FxI curve for the same cell model with inputs varying from 10 pA to 100 pA. The dashed lines indicate the minimum and maximum current amplitudes used to obtain the dynamic range of the cell.

The dynamic range of each cell in the sample was determined from its F-I curve (see Methods). To obtain the F-I curve of a cell model, we submitted it to somatic step current injections ranging from 10^1^ to 2.10^3^ pA in steps of 10 pA. In Figure 1A we show the voltage response of a cell from the SS group. The cell responds with a typical repetitive (tonic) firing of action potentials [24]. The F-I curve for the same cell is shown in Figure 1B together with its dynamic range. The firing frequency increases in approximately linear fashion with current amplitude up to a maximum frequency below 350 Hz.

**Figure 2:**
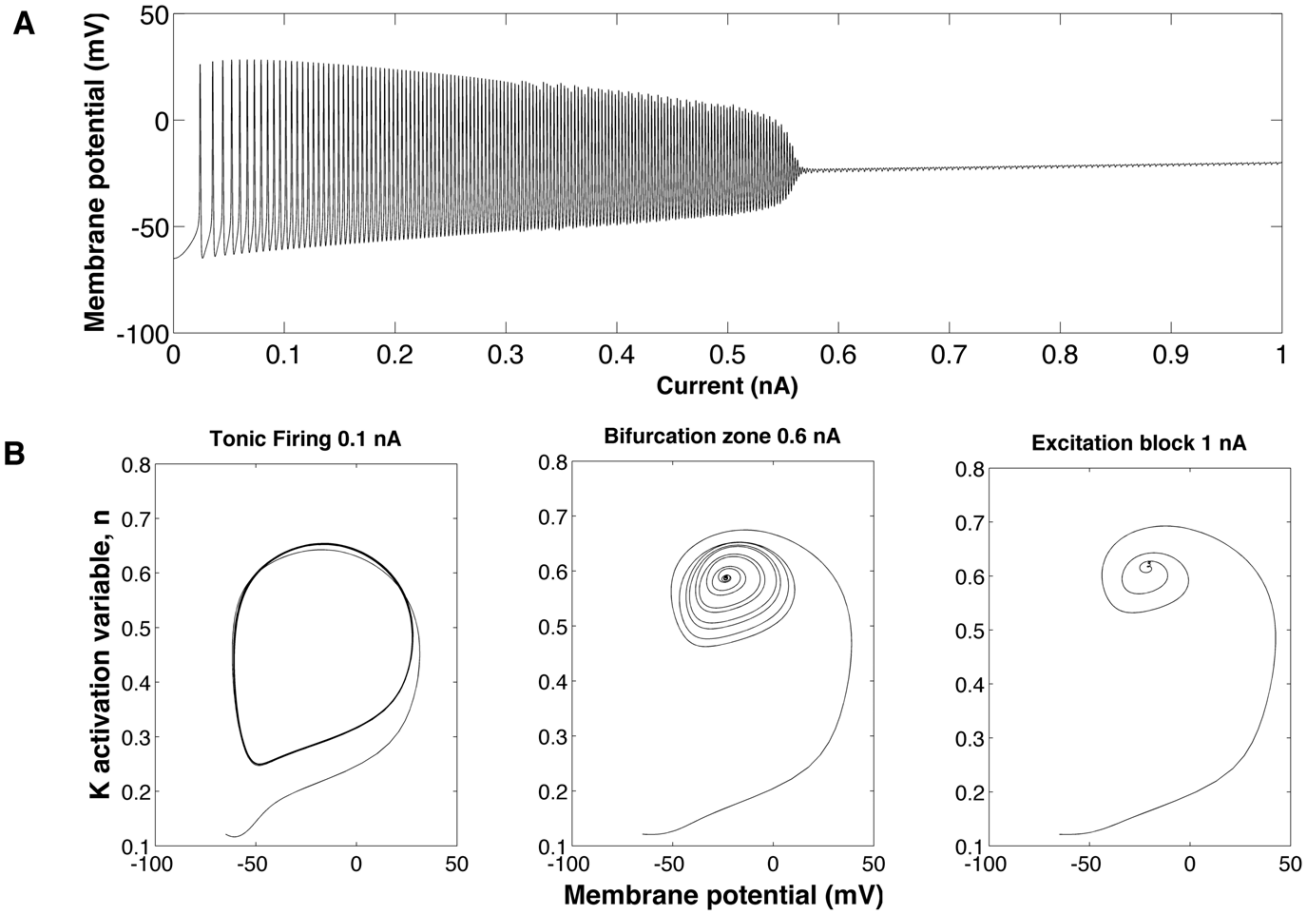
Transition from tonic firing to rest. (A) Amplitude of the action potential as a function of increasing current step. The current clamp is applied for 1000 ms and its amplitude increases linearly with time (I  = 0.01t). (B) Two-dimensional phase diagram showing membrane potential in the horizontal axis and the activation variable (*n*) of the potassion current for three different current clamp values: 0.1 nA (left), 0.6 nA (middle) and 1 nA (right). The left diagram shows a stable limit cycle while the right diagram shows a stable focus. The middle diagram corresponds to the region around bifurcation, in which there are many low amplitude oscilations before convergence to the fixed point.

The transition from tonic firing to rest observed in Figure 1B is typical of all ganglion cell models studied in this work. This behavior is also observed experimentally, where the tonic firing is blocked by injection of a depolarizing current above a threshold value [25]–[27]. The transition point corresponds to the cell’s physiological limit beyond which further increases in current amplitude may damage the cell. We have used dynamical systems analysis [28] to study this behavior, and the explanation can be understood with the help of Figure 2. Our analysis shows that the ganglion cell model undergoes a supercritical Andronov-Hopf bifurcation [29] as the current amplitude increases. When the model is stimulated with current clamp for 1000 ms, the amplitude of the spike gradually decreases with the current amplitude increase until it reaches the bifurcation point near 0.6 nA (Figure 2A). Phase portraits for the activation variable of the potassium current show the transition from limit cycle oscillations to a stable fixed point as the current crosses the critical point (Figures 2B).

**Figure 3:**
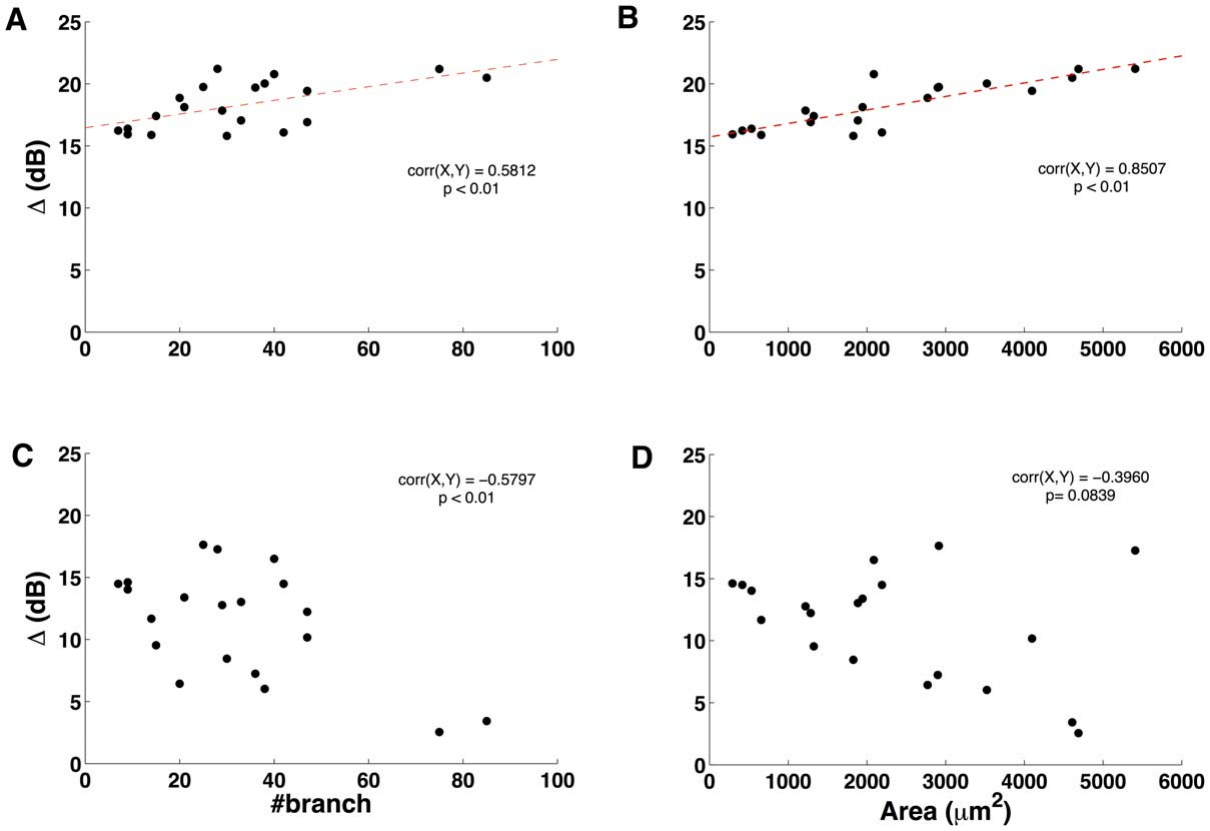
Scatter plots of dynamic range *versus* number of dendrites and total dendritic surface area for active and passive dendritic trees. Plots in the first row are for cells with active dendrites and plots in the second row are for cells with passive dendrites. The plots also show the Pearson correlation coefficients and p-values. (A) Scatter plot of dynamic range *versus* number of dendritic branches (active dendrites). The dashed line represents the best linear fit for the data (B) Scatter plot of dynamic range *versus* total dendritic surface area (active dendrites). The dashed line represents the best linear fit for the data. (C) Scatter plot of dynamic range *versus* number of dendritic branches (passive dendrites). (D) Scatter plot of dynamic range *versus* total dendritic surface area (passive dendrites).

Figure 3 gives the main results of our first study. It shows scatter plots of dynamic range *versus* number of dendritic branches (Figure 3A) and total dendritic surface area (Figure 3B) for the 20 cells in the sample. Figures 3C and 3D show the same scatter plots but for cells with passive dendrites. The dendrites were made passive by blocking all active channels of the cells’ dendrites.

A comparison between plots in the first row of Figure 3, for active dendrites, with plots in the second row, for passive dendrites, shows that active dendrites enhance the dynamic range of the cells. For passive dendrites, dynamic range values are always below 18 dB and can be as low as 3 dB while, for active dendrites, dynamic range values are always above 15 dB and can be as high as 21 dB. The mean, median and standard deviation for the dynamic range values are shown in Table 1.

**Table 1:** Dynamic range statistics for single ganglion cell models with active and passive dendrites.

	Mean (dB)	Median (dB)	SD (dB)
**Active**	18.25	17.98	1.93
**Passive**	11.29	12.49	4.41

We used the Wilcoxon rank-sum test to reject the null hypothesis of equal medians with p<0.001.

Figure 3 also shows that, for active dendrites, the two dendritic size measures considered by us, namely number of dendritic branches and total dendritic surface area, are positively linearly correlated with the dynamic range. The correlation is moderate with number of dendritic branches (Figure 3A) but strong with total dendritic surface area (Figure 3B). The linear regression lines for the two cases are shown in the plots and the angular coefficients are, *α* = 0.055 for the data in 3A and *α* = 0.001 for the data in 3B. These results suggest that active dendrites of large size enhance the dynamic range of single ganglion cells.

The positive correlation between dynamic range and size of the dendritic tree is lost when dendrites become passive. Figure 3C shows a moderate negative correlation between dynamic range and number of dendritic branches and Figure 3D shows a weak negative correlation between dynamic range and total dendritic surface area. This suggests that, in opposition to the active dendrites case, in the passive dendrites case large dendritic sizes may be detrimental to dynamic range.

The strong correlation between dynamic range and total dendritic surface area motivated us to do another experiment to investigate the effect of dendritic surface area alone on the dynamic range. Since dendritic surface area and number of dendritic branches are related, we designed an experiment in which only dendritic surface area varied while the number of dendritic branches remained constant. We chose a ganglion cell model from the SS group with passive parameters described in Table 2 and attached an extra dendritic compartment to its soma. Then we run two sets of experiments in which constant current steps of increasing amplitude were applied to the soma in the same fashion as in the experiments related to Figure 3. In the first set of experiments the extra compartment was passive, i.e. it had only leak conductance, and its surface area was equal to the somatic area multiplied by an area factor *A* that varied from 0.1 to 2. The second set of experiments followed the same protocol of the first but the extra compartment was now active, i.e. it had all conductances present in the other dendritic compartments with the same densities given in Table 3 (see Methods).

**Table 2:** Passive parameters of the ganglion cell models.

	Length (μm)	Diameter (μm)	Axial resistance (Ωm)	Leakage conductance density (mS/cm^2^)	Leakage reversal potential (mV)
Soma	-	-	110	8.10^−3^	−62.5
Axon	5340	1	110	8.10^−3^	−62.5
Initial segment	40	1	110	8.10^−3^	−62.5
Narrow segment	90	0.4	110	8.10^−3^	−62.5

**Table 3:** Maximum conductance densities of the active ion channels of the ganglion cell models.

Current type	Soma (mS/cm^2^)	Dendrites (mS/cm^2^)	Axon (mS/cm^2^)
Sodium	80	25	70
Calcium	1.5	2	0
Potassium	18	12	18
A-type inactivating potassium	54	36	0
Calcium dependent potassium	0.065	0.008	0.065

**Figure 4:**
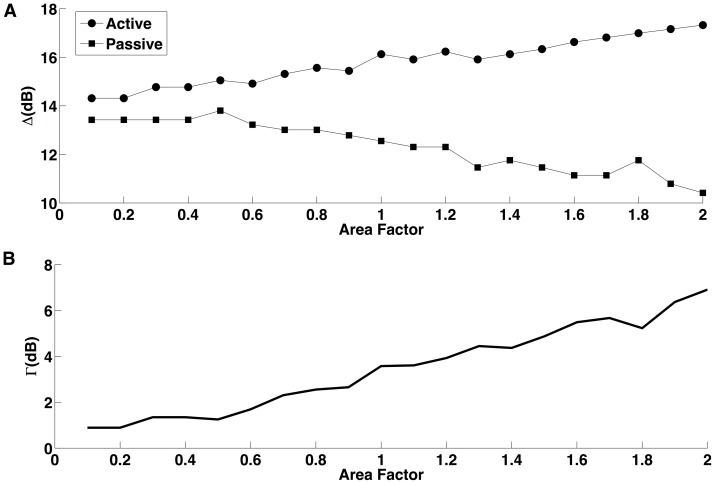
Effect of dendritic surface area on single cell’s dynamic range. (A) Dynamic range of the ganglion cell model as function of the area factor (see main text for a definition) of the extra attached compartment. Black dots indicate active extra compartment, and black squares indicate passive extra compartment. (B) Difference Γ between the dynamic ranges for active and passive cases as a function of the area factor.

The results of this experiment are given in Figure 4. Figure 4A shows plots of the cell’s dynamic range *versus* the area factor *A* for the cases in which the extra dendritic compartment is passive and active. The plots show that, for small areas (area factor ≤0.5), the dynamic range is only marginally larger when the compartment is active in comparison with when the compartment is passive. However, for larger areas the behaviors of the cases diverge dramatically: the dynamic range for the active case constantly increases with area while the dynamic range for the passive case constantly decreases with area. The difference Γ between the dynamic ranges for the active and passive cases is shown in Figure 4B as a function of the area factor. For an increase in the area factor from 0.5 to 2, Γ increases by 6.5 dB. This result shows that, for active dendrites, increases in dendritic surface area alone are capable to enhance the dynamic range of a single ganglion cell.

**Figure 5:**
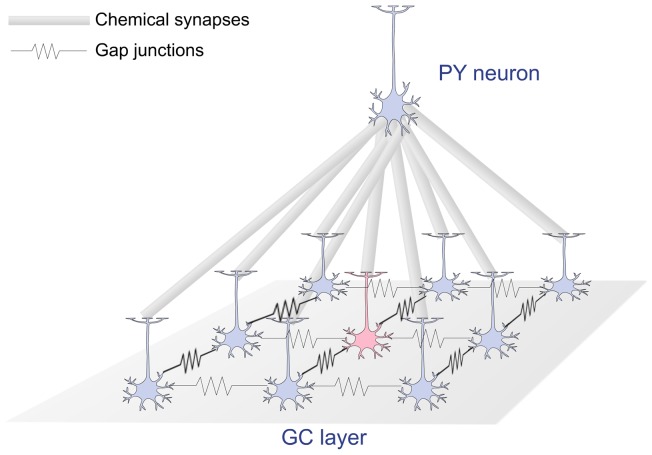
A scheme of the network model. Ganglion cells are placed in the vertices of a 3×3 square grid and are coupled with their first neighbors via dendrodendritic gap junctions. Each ganglion cell makes an excitatory chemical synapse with a pyramidal cell from the LGN. Only the central cell of the array (indicated by an arrow) receives external input in the form of current clamps of varying amplitudes.

The second study was designed to assess the effect of electrical coupling between ganglion cells on the collective dynamic range of the cells. We simulated a 3×3 square array of ganglion cells coupled via dendrodendritic gap junctions as shown in Figure 5 (for details see Methods). All cells in the array were taken from the SS group. Only the ganglion cell positioned at the center of the array received external inputs while the other passively received stimuli through the gap junctions. The ganglion cells were also coupled by chemical synapses to a single neuron (also shown in Figure 5), which represents a pyramidal neuron from the lateral geniculate nucleus (LGN) of the thalamus. This pyramidal neuron was modeled as a single-compartment cell [30] (see Methods).

**Figure 6:**
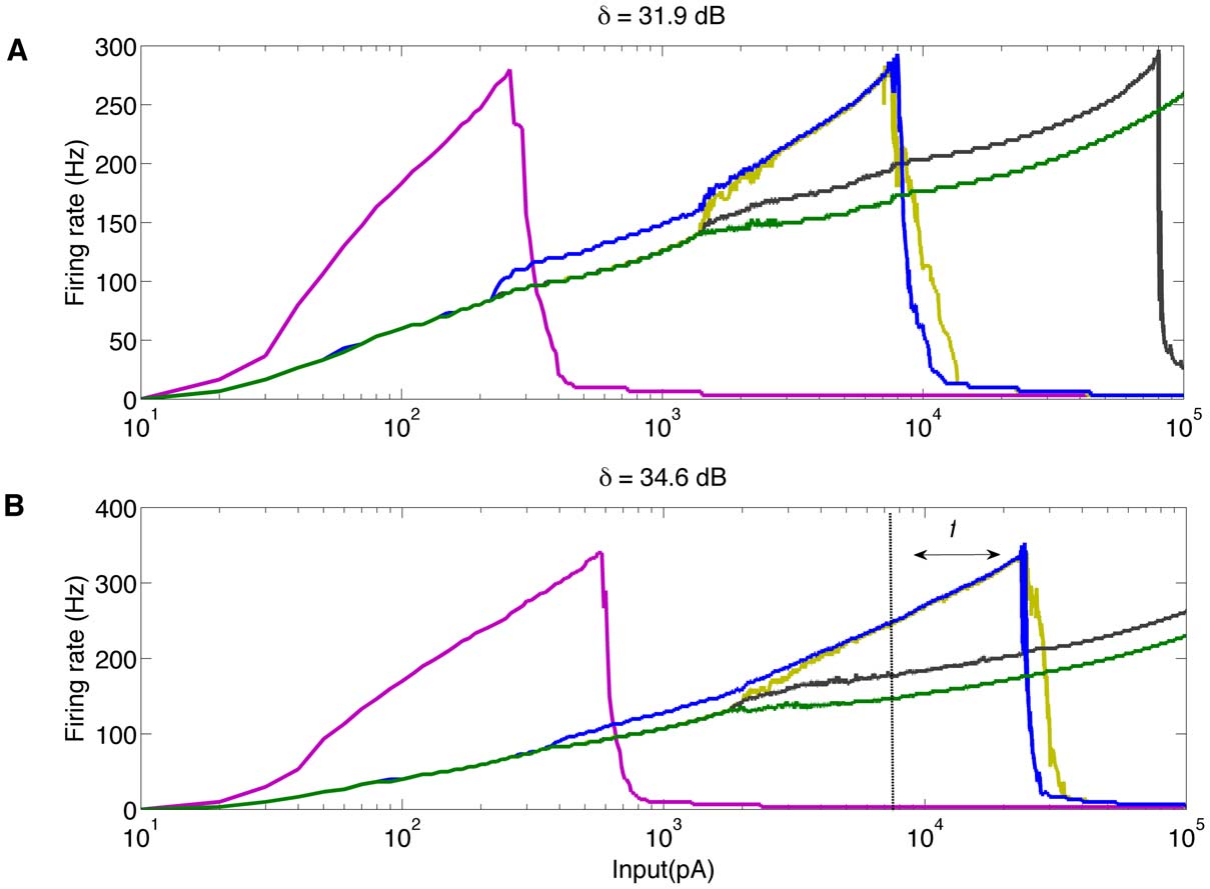
F-I curves for ganglion cells in the array. In all cases, to calculate steady state firing frequencies, the input current was applied for 0.3 seconds. (A) Ganglion cells with passive dendritic trees. (B) Ganglion cells with active dendritic trees. The dashed line represents the maximum current value for the cell with response curve indicated in blue in Figure A. The factor *f* shows the displacement of this maximum current to the right when cells have active dendrites. The average dynamic range of the 9 ganglion cells of the network (δ) for each case is shown above the corresponding graph.

In our experiments, step current inputs were applied to the central neuron of the array with amplitudes varying from 10^1^ to 10^5^ pA. This central neuron excites the other neurons via gap junctions so that we can relate the steady state firing frequency of each one of the nine neurons in the array to the amplitude of the applied current input. Figure 6 shows the responses of the ganglion cells in the array for the range of input currents used. Figure 6A shows responses for cells with passive dendrites and Figure 6B shows responses for cells with active dendrites.

Each coupled cell exhibits a F-I pattern similar to the one of the single cell in Figure 1, with firing rate increasing as a function of the stimulus amplitude until the physiological limit is reached and the cell is not able to spike anymore. In both figures 6A and 6B, the leftmost curve is the F-I curve of the central cell. This cell has the steepest F-I curve slope and reaches the physiological limit for a current amplitude well below the amplitudes that put the other cells at physiological limit. For some cells, the maximum current amplitude is even beyond the range of input values used to plot the graphs. As a consequence of this, the range of current amplitudes for which the coupled cells can respond before saturating is enhanced in comparison with the uncoupled cell case. The dynamic range of the single (active) cell (Figure 1B) is 16.9 dB and the dynamic range of the coupled cells is 31.9 dB when cells are passive (Figure 6A) and 34.7 dB when cells are active (Figure 6B).

Although the enhancement was similar for the simulations with passive and active trees, the maximum dynamic range was obtained for the coupled array of ganglion cells with active dendrites (Figure 6B). This result suggests that although coupling by gap junctions may play a major role in the enhancement of the dynamic range of the ganglion cell layer of the retina, intrinsic ionic membrane mechanisms may also contribute to the enhancement.

A quantitative estimate of the contribution of active dendrites to the further enhancement of the dynamic range of a cell in the coupled array is provided by factor *f* given in Figure 6B. The factor *f* was calculated as I_A_/I_P_, where I_A_ is the maximum current amplitude for the indicated cell of the coupled array with active dendrites and I_P_ is the maximum current amplitude for the same cell with passive dendrites. The calculated value of *f* = 2.94 shows that when cells in the coupled array have active dendrites the chosen cell can respond to inputs almost 3 times higher before saturation when compared to the same cell in the same coupled array but with all cells in the array with passive dendrites.

**Figure 7:**
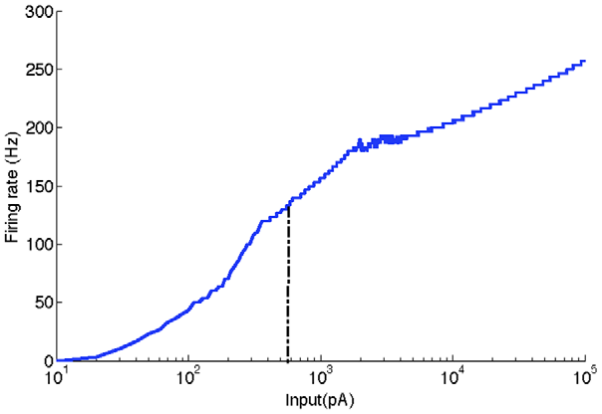
F-I curve of the pyramidal neuron model. Firing frequency of the pyramidal neuron of the LGN as a function of input current applied to the central cell of the ganglion cell array. The vertical dashed line gives the input current for which the central ganglion cell in the array stops firing (580 pA). The dynamic range of the FxI curve is 34.8 dB.

An indirect way to assess the dynamic range of the coupled array of ganglion cells is by measuring the dynamic range of the pyramidal neuron that receives input from them. Figure 7 shows the steady state firing frequency of this pyramidal neuron as a function of the amplitude of current injected in the central cell of the coupled ganglion cell array (only the case of active cells was considered). For comparison, the vertical dashed line shows the maximum current amplitude supported by the uncoupled central ganglion cell of the array. This current amplitude gives the upper limit of the range of input currents applied to the central ganglion cell of the array to which the pyramidal neuron responds when ganglion cells are uncoupled. Figure 7 shows that when ganglion cells are coupled this upper limit is displaced more than two orders of magnitude to the right.

## Discussion

The main result of our simulation studies with morphologically reconstructed ganglion cell models is that active dendrites enhance the dynamic range of the cells. The other two important results are that (1) for isolated ganglion cells, the dynamic range has positive and significant correlation with the size of the active dendritic tree (the correlation is stronger with total dendritic surface area than with number of dendritic branches), and (2) by coupling ganglion cells via gap junctions the dynamic range of the coupled cells is further enhanced, being greater than the average dynamic range of the individual cells.

Why a cell with active dendrites has a larger dynamic range than a morphologically similar cell with passive dendrites? Passive dendrites act as current sinks. Current injected at the soma escapes to dendrites reducing the efficiency of the input current in making the cell fire. Active dendrites, on the other hand, allow soma-generated spikes to propagate into the dendritic tree. These in turn generate dendritic spikes, which interact nonlinearly across the dendritic arbor leading to creation and annihilation of spikes and the consequent enhancement of the cell’s dynamic range [10].

This explains the lower values of dynamic range for ganglion cells with passive dendrites in comparison with ganglion cells with active dendrites. It also explains the positive correlation between dynamic range and size of the dendritic tree for cells with active dendrites and the negative correlation between dynamic range and size of the dendritic tree for cells with passive dendrites. Since passive dendrites act as current sinks, the larger the dendritic tree, the more space for current to sink. On the other hand, larger dendritic trees have more active ion channels to support spike creation and summation.

And why dendritic surface area correlates better with dynamic range than with number of dendritic branches? The stronger correlation of dynamic range with dendritic surface area than with number of dendritic branches means that dendritic surface area is a better predictor of dynamic range than number of dendritic branches. As commented above, the critical factor for the enhancement of the dynamic range of a cell is to have active ion channels distributed over its dendrites. So, surface area correlates better with dynamic range simply because it is a better estimator of the number of ion channels in a dendritic tree than number of dendrites. This is because the number of ion channels in a dendritic tree is determined by channel densities *per area* rather than *per dendrite*.

A demonstration that the key factor to enhance the dynamic range of a ganglion cell is the number of active ion channels in its dendrites is given by the results shown in Figure 4. In that study, the number of dendritic branches and, therefore, the complexity of the dendritic tree, was kept constant and only the surface area of a dendritic compartment attached to the soma varied. When this compartment had only passive leakage channels, increases in its area lead to decrements in the dynamic range (surface area and dynamic range were negatively correlated). But when the compartment had active ion channels, increases in its surface area corresponded to increases in dynamic range (the two were positively correlated). Since the active ion-channel conductance densities were kept constant during these experiments, increases in surface area corresponded to increases in the absolute number of active ion channels.

Our results show that when ganglion cells are coupled by gap junctions their dynamic range is much higher than the average dynamic range of isolated ganglion cells. Even ganglion cells with passive dendrites, when coupled by gap junctions, have a larger dynamic range than isolated ganglion cells with active dendrites. The average dynamic range of these latter is 11.30 dB and the dynamic range of the former is 31.9 dB. When the coupled cells have active dendrites, the dynamic range of the array is a little higher: 34.7 dB.

Why coupled cells with passive dendrites have a larger dynamic range than isolated ganglion cells with active dendrites? The reason for this is that dendrodendritic coupling by gap junctions interlinks the somata of the cells, which have active ion channels, transforming the system into a spatially extended excitable medium. The mechanism responsible for the enhancement of the dynamic range of the coupled ganglion cells with passive dendrites is, therefore, the same one responsible for the enhancement of the dynamic range of an isolated ganglion cell with active dendrites, namely nonlinear summation of spikes [10], [15]. And the dynamic range is enhanced even more when the dendrites of the coupled cells have active conductances themselves.

Based on our results on the dynamic range of coupled ganglion cells we can make two predictions: (1) blockade of gap junctional coupling of ganglion cells in the vertebrate retina should strongly reduce (approximately by 40%) the output dynamic range of the retina; and (2) selective suppression of dendritic (but not somatic) spiking of coupled ganglion cells in the vertebrate retina should reduce the output dynamic range of the retina by a much smaller factor (approximately 9%). These reductions could be verified by simultaneous recording from lateral geniculate nucleus pyramidal cells.

In a previous work, we used a detailed model of the scotopic pathways that convey information from rods to a single ganglion cell of the vertebrate retina to study the effect of coupling by gap junction at the first stages of these pathways on the dynamic range of the ganglion cell [20]. In this work, we used morphologically detailed ganglion cell models to extend our early work and investigate dynamic range-enhancing mechanisms at the final stage of retina. The combined results of our present work imply that the largest possible dynamic range achievable at this final stage is occurs when ganglion cells with large and active dendritic trees are coupled by gap junctions. We propose that these are the structures more likely to preserve the large dynamic ranges achieved at earlier processing stages in the vertebrate retina.

We further predict, based on our results, that it is unlikely that any type of ganglion cell would have a passive tree or a very low channel density at the dendrites. We also predict that, if there are uncoupled ganglion cells in the retina, these are distributed over the retina so that cells with large dendritic trees (which imply large dynamic ranges) are able to integrate signals from circuits mediated by rods and cones responding to dim and bright light conditions. On the other hand, ganglion cells with small dendritic trees should be specialized to photopic or scotopic conditions. These predictions could be experimentally confirmed in the future with a detailed study on the distribution of morphologically distinct ganglion cells over the vertebrate retina.

Future investigations can provide a better understanding on the roles of cell connectivity and membrane properties on the dynamic range of the retina. The model can be further improved with a more realistic synaptic input distribution over the dendrites of ganglion cells and also extended to include the main circuits involved with dim and bright light processing in the retina.

## Methods

### Single cell models

We worked with a sample of 20 morphologically reconstructed, three-dimensional ganglion cell models from the tiger salamander (*Ambystoma tigrinum*) retina [23]. The models are available at modelDB (http://senselab.med.yale.edu/modelDB). The reconstructed neurons were classified into four groups: medium-complex (MC), medium-simple (MS), small-complex (SC), and small-simple (SS) [24]. Five different neurons were taken from each group totalizing 20 neurons with distinct morphologies. In addition to the reconstructed dendritic tree, each model includes an axon with an initial and a narrow segment. The passive parameters that are common to all cells are described in Table 2. The length and diameter values for the soma are not given in Table 2 because they are based on cell morphology data and have a different value for each model.

The same set of active ion channels were placed in all ganglion cell models. Each model has four voltage-dependent channels (Na, Ca, K, and K_A_), one calcium-dependent channel (K_Ca_). The dynamics and parameters of the calcium current were able to fit the high-voltage activated component of the calcium current (L-type) described in a previous experimental work [31]. The K channel simulates the classical delayed rectifier potassium current and was modeled with no inactivation kinetics while the Na and K_A_ channels have inactivation kinetics. The channel densities for each group of compartments are given in Table 3.

To obtain the F-I curve of an isolated ganglion cell we submitted it to steps of somatic current clamp of fixed amplitudes. The duration of each step was 300 ms and the current amplitudes varied from 10 pA to 1000 pA. The duration of 300 ms was chosen because, based on our studies, it is sufficient for a reliable estimate of the steady state firing frequency of a ganglion cell to a step current.

For all simulations, the dynamic range (Δ) was calculated as:
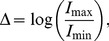
(1)where *I*_min_ is the stimulus value for which the response reaches its minimum value and *I*_max_ is the stimulus value for which the response reaches its maximum value. The value Δ represents the range of inputs that can be coded by the cell before its saturation [15]. As exemplified in Figure 1B, Δ is calculated in dB where the only information necessary are the lowest input that is capable of generate at least a single spike in the model and the highest input before the response maximum.

The single compartment model of a lateral geniculate nucleus pyramidal neuron used to obtain Figure 7 was taken without changes from a previous work [30]. It contains a slow voltage-dependent K current, the I_Na_ and I_K_ currents and the T-type calcium current. The values of the parameters used to model these channels can be seen at the original work [30].

### Synaptic connections and network topology

We used experimental evidence of dendrodendritic bidirectional gap junctions connecting ganglion cells [21]–[23] to simulate the electrical coupling between two neighboring cells as a single resistance with symmetrical conductance of 1.35 nS [21]. Since the exact distribution of gap junctions over the dendritic tree is unknown, in all simulations the gap junction connection between two cells linked their primary dendrites.

The excitatory chemical synapse between the axon of a ganglion cell and the LGN pyramidal cell was modeled by a closed/open gating scheme (*t*_binding_  = 0.2 ms, *t*_unbinding_  = 1.1 ms), activated by square-wave transmitter pulses of amplitude 1 mM and duration 0.3 ms [32]. The reversal potential of the synapse was 0 mV and the maximal conductance was adjusted to 600 pS. All synapses connecting ganglion cells to the pyramidal cell were modeled with these parameters and dynamics.

The network model consisted of 9 ganglion cells and a single LGN pyramidal cell. The ganglion cells were arranged in a 3×3 square grid and connected by gap junctions as shown in Figure 5. The 9 ganglion cells make excitatory synapses to the pyramidal neuron (Figure 5). In each simulation, only the ganglion cell located at the center of the 3×3 array was stimulated by the current clamp. The current was injected at the cell’s primary dendrite. The 9 ganglion cells used to construct the array were randomly chosen from the SS group. The same group of 9 cells was used in all simulations. Cells were chosen from the SS group because of their small dynamic ranges when uncoupled and to reduce the computational cost of the simulations.

To obtain the average response of the ganglion cells and the pyramidal neuron for a given amplitude of current clamp, we stimulated the network with the current for 0.3 seconds and counted the number of spikes during this period. We used current amplitudes in the range from 10^1^ pA to 10^5^ pA separated by steps of 10 pA. We consider 0.3 seconds a period sufficiently long for a reliable estimate of the ganglion cells’ firing frequency and to obtain the dynamic range of the ganglion cells and the pyramidal neuron. The simulations were performed in NEURON 7.1 [33], [34] and the numerical integration of the equations was performed using the backward Euler method with a time step of 0.1 ms.
